# Predicting Adolescent Adjustment and Well-Being: The Interplay between Socio-Emotional and Personal Factors

**DOI:** 10.3390/ijerph16234650

**Published:** 2019-11-22

**Authors:** Usue de la Barrera, Konstanze Schoeps, José-Antonio Gil-Gómez, Inmaculada Montoya-Castilla

**Affiliations:** 1 Department of Personality, Assessment and Psychological Treatment, Faculty of Psychology, University of Valencia, 46010 Valencia, Spain; usue.barrera@uv.es; 2 Department of Psychology, Faculty of Health Sciences, European University of Valencia, 46010 Valencia, Spain; konstanze.schoeps@universidadeuropea.es; 3 Instituto Universitario de Automática e Informática Industrial, Universitat Politècnica de València, 46022 Valencia, Spain; jgil@upv.es

**Keywords:** adolescence, emotional competencies, self-esteem, psychological adjustment, subjective well-being, regression models, fsQCA models

## Abstract

Social and emotional factors such as emotional competence and self-esteem are protective factors that promote adolescent mental health and well-being. In this paper, we will examine the combined contribution of these socio-emotional factors in addition to personal factors, in the prediction of psychological adjustment and subjective well-being in adolescence. The study included 840 adolescents aged between 12 and 16 years old (*M* = 13.37, *SD* = 1.16, 51.4% girls). We measured personal variables (sex, age, number of siblings), socio-emotional variables (emotional competence and self-esteem), psychological adjustment (emotional and behavioural problems) and subjective well-being (life satisfaction and affect balance). Besides descriptive analysis and Pearson bivariate correlations, two different methodologies were performed, including hierarchical regression models and a fuzzy-set qualitative comparative analysis (fsQCA). The results show that emotional competence is a protective factor for optimal adjustment and well-being, and suggest that self-esteem reinforces this relation. Different patterns were observed for female and male adolescents of different ages and with different family backgrounds. The practical implications of our findings for intervention programs have been discussed.

## 1. Introduction

Adolescence is a period of physical and psychological development involving neurocognitive, affective, social, and academic changes [1]. Many adolescents have difficulties adjusting and coping with these challenges, which may impact on their mental state, increasing the risk of long-term mental health issues [2]. Indeed, seventy-five percent of mental disorders are estimated to occur for the first time before the age of 25 [3,4]. Furthermore, adolescents perceive lower levels of life satisfaction and experiment emotional distress more frequently and with greater intensity than younger children or older adults [5,6]. Adolescence is therefore a developmental stage with high risks in terms of psychological problems, subjective well-being, and adjustment [7,8].

Psychological adjustment refers to a young person’s ability to adapt adequately to his or her environment, considering emotional, behavioural, and social aspects [9]. An inappropriate adjustment to the immediate social context increases behaviour problems, such as substance abuse, aggression, violence, and delinquency [10]. Furthermore, psychological maladjustment increases the probability of presenting emotional problems (e.g., feelings of distress, fears, and worries) and somatic complaints (e.g., headache and stomach pain) [11]. In addition to the risk of problems arising from lack of adjustment, there is often a decline in subjective well-being during adolescence [12].

Subjective well-being is composed of a cognitive dimension: life satisfaction, and an affective dimension: positive and negative affects [13,14,15]. Both components are related to a variety of positive aspects relevant to adolescents’ lives, such as social support, self-esteem, self-efficacy, and optimism [16,17]. In this sense, life satisfaction and positive affect are positively related to self-esteem, while negative affect is inversely associated with self-esteem [16]. Studies have shown that enhancing adolescent well-being also prevents the onset of psychopathology [18] and is related to optimal levels of personal, social, and emotional functioning [19]. 

Adolescence is often seen as a period of vulnerability in which psychological adjustment and well-being may be at risk, but it is also associated with greater brain plasticity [20]. Indeed, young brains are easily shaped through experience, due to the human brain’s ability to mould itself. The neural plasticity of the teen brain allows adolescents to learn things faster and makes their memories more robust. This makes it the perfect time for developing their strengths [21]. Traditional research in psychology has focused on the risk factors that may have a negative impact on adolescent mental health [22]. However, in the field of positive psychology there is a need to focus on adolescents’ strengths in order to prevent the onset of health problems [23]. Instead of focusing and trying to prevent adolescents’ problems, this paradigm therefore considers the strengths, competences, and resources that maximize young people’s healthy development and well-being [24,25].

There are socio-emotional factors that may protect adolescents’ health and well-being. One of the emotional strengths that has been studied in association with psychological adjustment and well-being is emotional intelligence [26,27,28]. Emotional intelligence, i.e., the ability to identify, understand, and regulate emotions [29], has a positive effect on cognitive and emotional development during childhood and adolescence [30]. Emotional intelligence is considered to be part of a broader concept, called emotional competence. While emotional intelligence comprises only the skills of emotional information processing, emotional competence assumes also the significant contribution of individual development in a social-cultural context [31]. Indeed, recent studies have shown the benefits of emotional-skill training through effective intervention programs. Emotional competence learning apparently not only reduces the incidence of emotional problems, but also fosters well-being and mental health [32,33,34]. 

Self-esteem is another important socio-emotional factor that influences psychological adjustment and well-being in adolescence [35,36,37]. Adolescent self-esteem is very fragile and susceptible to internal and external changes [38,39]. However, positive self-esteem could have an important impact on young people’s health and well-being. For instance, adolescents with high self-esteem are able to adapt to stressful life events [40,41,42]. Recent studies suggest that future problems may be alleviated by increasing adolescents’ self-esteem, including extreme behaviours such as suicide attempts [43].

In addition to these socio-emotional protective factors, there are also personal characteristics that may affect adolescent adjustment and well-being, such as sex and age. As regards psychological adjustment, boys present more behavioural problems [44]; whereas girls tend to experience more emotional symptoms [45]. In terms of age, young people before puberty (pre-adolescents) adjust more easily to internal and external changes than adolescents [46,47]. Subjective well-being also appears to be influenced by sex and age, with girls scoring higher than boys for self-reported life-satisfaction, and scores for both tending to decline with age [48]. The number of siblings seems to be another relevant but neglected factor regarding youth development [49]. A large number of siblings appears to be related to better social functioning in adolescents with autism spectrum disorder [50]. In healthy children, those with more siblings (five or more siblings) appear to have more emotional abilities such as empathy than those who have fewer siblings (one or two) [51]. Although studies suggest that siblings are crucial for adolescent development, very few studies analysing the influence of sibling numbers on adolescent adjustment and well-being in the normal population have been conducted. 

Drawing from previous research, there are socio-emotional factors such as emotional competence and self-esteem that predict either adolescent adjustment or well-being [27,28,37]. Most studies usually focus instead on preventing psychological problems such as behavioural and emotional difficulties [36] or consider the positive approach of promoting mental health and well-being [52]. To our knowledge, there are few studies that integrate both perspectives in order to simultaneously determine potential risk and protection factors for psychological adjustment and subjective well-being. Furthermore, personal characteristics such as sex and age have been widely studied to determine their influence on well-being, and health outcomes have been firmly established [45,48]. However, the study of other personal factors such as the number of siblings has been limited [51]. This study focuses not only on adjustment problems, but also on subjective well-being; it includes socio-emotional factors as well as personal characteristics; and it incorporates two types of methodology: linear regression models and the fuzzy-set qualitative comparative analysis (fsQCA). The first strategy is based on linear models and focuses on the individual contribution of each variable, whereas the second strategy, qualitative comparative analysis (QCA), enables an in-depth analysis of how causal conditions contribute to an outcome. QCA assumes that the influence of a particular attribute on a specific outcome depends on a combination of attributes rather than on individual levels of the attributes [53].

The purpose of this study was therefore to examine the combined contribution of the socio-emotional factors of emotional competence and self-esteem, and the personal factors of sex, age, and number of siblings, in the prediction of psychological adjustment and subjective well-being in adolescence. Based on the research approaches mentioned above, we hypothesized that (1) higher emotional competence and higher self-esteem will predict fewer behavioural problems and fewer emotional symptoms, indicating better adjustment; (2) higher emotional ability and higher self-esteem will predict higher life satisfaction and affect balance, indicating more well-being; (3) girls will experience more emotional symptoms than boys, whereas boys will report more behavioural problems than girls―as well as the fact that younger adolescents might be better adjusted and have more well-being than older ones; and (4) that the number of siblings will be associated with better adjustment.

## 2. Materials and Methods

### 2.1. Participants

The study included 840 adolescents aged between 12 and 16 years old (*M* = 13.37, *SD* = 1.16, 51.4% girls) from 6 public and private high schools in the autonomous communities of Madrid and Valencia (Spain). The sample was chosen intentionally with the following inclusion criteria: (a) the school’s interest in collaborating with the research group; (b) the students’ age was between 12 and 16 years; (c) the students had their parents’ or guardians’ signed consent to participate in the research. The adolescents belonged to the first, second, third, and fourth grade of compulsory secondary education: 41.7% (*n* = 350) were first year students (girls: 49.1%; boys: 50.9%); 35.1% (*n* = 295) were second year students (girls: 50.2%; boys: 49.8%); 12.1% (*n* = 102) were third year students (girls: 53.9%; boys: 46.1%); and 11.1% (*n* = 93) fourth year students (girls: 61.3%; boys: 38.7%). As regards siblings, 20.7% of the adolescents were only children, 57.9% had one sibling, 14.2% had two siblings, 4.9% had three siblings, and 2.4% had 4 or more siblings.

### 2.2. Variables and Instruments

#### 2.2.1. Personal Variables

Personal or socio-demographic data regarding the students’ age, sex and number of siblings were collected using ad hoc questions. 

#### 2.2.2. Socio-Emotional Variables

Emotional competencies. Emotional skills were evaluated by the Emotional Skills and Competence Questionnaire [54] reduced and validated for the Spanish population (ESCQ-21) [55]. The questionnaire evaluates emotional competence from an ability perspective, contains 21 items, and is answered on a 6-point Likert scale (1 = Never; 6 = Always). The instrument consists of three scales: Perception and Understanding (α = 0.82; e.g., “When I meet an acquaintance, I immediately notice his/her mood”), Labelling and Expression (α = 0.91; e.g., “I am able to express how I feel”) and Management and Regulation (α = 0.78; e.g., “I try to keep up a good mood”). The questionnaire has shown good reliability in this sample.

Self-esteem. Self-esteem was assessed using the Spanish version of the Rosenberg Self-esteem Scale (RSE) [56,57] which consists of 10 items scored from 0 (Strongly disagree) to 4 (Strongly agree) points. The reliability index of the scale was adequate in this sample (α = 0.88).

#### 2.2.3. Psychological Adjustment

Emotional and behavioural adjustment. The Strengths and Difficulties Questionnaire (SDQ) [9] evaluates emotional and behavioural constructs. The Spanish version of the SDQ [58] was used to assess adolescents’ emotional and behavioural problems. It is a scale with 5 factors, scored from 0 (Not True) to 2 (Certainly True), but for the purposes of this study we used the two subscales of Emotional Symptoms (α = 0.68) and Behavioural Problems (α = 0.53). The critical value of Cronbach’s alpha is α > 0.50 [59] and as such the reliability of the scale is considered acceptable. In the self-report version of the SDQ, a score above 20 on the global Difficulties scale indicates the presence of discomfort and/or presence of pathological symptoms, a score between 16 and 19 is considered at the limit, and a score below 15 is a normal state with no difficulties. 

#### 2.2.4. Subjective Well-Being

Satisfaction with life. Life satisfaction was measured by the Satisfaction With Life Scale (SWLS) [47,60]. The instrument evaluated people’s satisfaction with their living conditions. The scale is composed of five items, and is answered on 7-point Likert scale (1 = Completely disagree; 7 = Completely agree; e.g., “In most ways my life is close to my ideal”). This scale also shows good psychometric properties [61]; in this study (α = 0.84).

Affect Balance. Affectivity was assessed using the Scale of Positive and Negative Experiences (SPANE) [62]. The instrument is composed of 12 items, six of which refer to positive experiences (e.g., “In the last 4 weeks I’ve had positive feelings”) and six of which refer to negative experiences (e.g., “In the last four weeks I’ve had negative feelings”). People are questioned about how often they have experienced positive and negative feelings over the past 4 weeks, and they answer on a five-point scale (1 = Never, 5 = Always). A global affect balance can be obtained by subtracting the negative affect score from the positive affect score. Reliability in positive affect (α = 0.87) and negative affect (α = 0.77) was adequate in this sample.

### 2.3. Procedure

First, the ethics committee of the University of Valencia and the Ministry of Education, Culture and Sport gave their approval and authorization for the study (H152865096049) and the research team contacted the high schools. The parents were then informed about the content of the study and they signed an informed consent form. The adolescents completed the questionnaires using LimeSurvey during their usual school hours, in about forty-five minutes. Adolescents whose parents did not sign the consent form were excluded from the study.

### 2.4. Data Analysis

First, descriptive analysis and Pearson bivariate correlations were produced to estimate the relationship between variables. Next, the effects of age, sex, number of siblings, emotional competencies, and self-esteem on emotional and behavioural adjustment and well-being were analysed using two different methodologies: hierarchical regression models and a fuzzy-set qualitative comparative analysis (fsQCA).

Four hierarchal regression models were run for each indicator of psychological adjustment (emotional symptoms and conduct problems) and well-being (life satisfaction and affect balance). In all four models, predictors were entered in three blocks in order of their importance in predicting the criterion variable: (1) demographic variables: sex, age and number of siblings, (2) all three dimensions of emotional competence, and (3) self-esteem. Assumptions of no multicollinearity, homoscedasticity, and independent errors were tested previously to conducting the regression analyses.

The calibration values for performing a fuzzy-set qualitative comparative analysis (fsQCA) were calculated. The raw data responses were transformed into fuzzy-set responses. All the missing data were removed, and the constructs (variables) were obtained by multiplying their item [63]. The values of each variable were then recalibrated considering the three thresholds: percentile 10 (low agreement or fully outside the set), percentile 50 (intermediate level of agreement, neither inside nor outside the set), and percentile 90 (high agreement or fully in the set) [64]. All values of all variables had to be between 0 and 1. The sociodemographic variables were calibrated manually. The age and number of siblings were coded according to their values in five points equidistant between 0 and 1, and for gender, 0 was coded as a girl and 1 as a boy. Descriptive analyses of the variables studied were also carried out. Finally, necessary and sufficient conditions tests assessed the effect of the sociodemographic variables, emotional competences and self-esteem on high levels of maladjustment and subjective well-being. Fs/QCA 2.5 software (University of California, Irvine, CA, USA) was used to perform the analysis.

## 3. Results

### 3.1. Descriptive Analysis and Relationships between Emotional, Cognitive and Personal Factors

The correlations between the study variables (Table 1) indicated that age was negatively related to emotional competencies, self-esteem, and well-being (*r* between −0.07 and −0.16), while the associations with emotional and behavioural problems is positive, with only small but significant correlation coefficients (*r* between 0.09 and 0.16). The three dimensions of emotional competence (perceive, express, and manage emotions) demonstrated significant and positive correlations with self-esteem, life satisfactions, and affect balance, with moderate-high effect size (*r* between 0.15 and 0.58). Smaller and negative correlation coefficients were observed in the relation between emotional competence and emotional and behavioural problems (*r* between −0.07 and −0.37). Self-esteem correlated significantly and in a positive way with well-being (*r* between 0.62 and 0.63), whereas the associations with adjustment problems were negative (*r* between −0.28 and −0.58). Interestingly, the highest correlation coefficients were observed in these relationships. Lastly, the association of both dimensions of well-being, life satisfaction, and affect balance, with emotional and behavioural problems was significant and negative (*r* between −0.26 and −0.53).

### 3.2. Emotional and Personal Predictors of Adjustment and Well-Being

Predictive analysis of study variables was performed by four hierarchal regressions (Table 2), one for each indicator of psychological adjustment (emotional symptoms and behavioural problems) and well-being (life satisfaction and affect balance).

The prediction of adjustment was carried out on the one hand with emotional symptoms and on the other, with behavioural problems. In the prediction of emotional symptoms, three steps were established in the model (*R*^2^ = 0.38; *p* < 0.001). In the first step, demographic variables were entered to estimate the impact of sex, age, and number of siblings (*ΔR*^2^ = 0.09; *p* < 0.001). In the second step, the three dimensions of emotional competence were entered (*ΔR*^2^ = 0.12; *p* < 0.001), followed by the entry of self-esteem in the third step (*ΔR*^2^ = 0.16; *p* < 0.001). The final model therefore indicates that sex (β = −0.14; *p* < 0.001), age (β = 0.07; *p* < 0.01), perceived emotions (β = 0.11; *p* < 0.001), managed emotions (β = −0.10; *p* < 0.01) and self-esteem (β = −0.50; *p* < 0.001) may predict levels of emotional symptoms in a negative and significant manner.

For the prediction of behavioural problems, the model consisted of three steps (*R*^2^ = 0.10; *p* < 0.001). First, sex, age, and number of siblings were entered in the equation (*ΔR*^2^ = 0.02; *p* < 0.001). In the next step, all three emotional abilities were added to the equation (*ΔR*^2^ = 0.05; *p* < 0.001). In the third step self-esteem was included (*ΔR*^2^ = 0.04; *p* < 0.001). In the final model, sex (β = 0.15; *p* < 0.001), number of siblings (β = 0.07; *p* < 0.05), managing emotions (β = −0.12; *p* < 0.01) and self-esteem (β = −0.24; *p* < 0.001) appeared to be the significant predictors of behavioural problems.

The prediction of subjective well-being was made on the one hand based on life satisfaction and, on the other, based on affects. The prediction model of life satisfaction was the same manner as the other previous models (*R*^2^ = 0.45; *p* < 0.001). All three demographic variables were therefore entered as the first block (*ΔR*^2^ = 0.04; *p* < 0.001), followed by all three dimensions of emotional competence in the second block (*ΔR*^2^ = 0.29; *p* < 0.001) and finally self-esteem in the third and last block (*ΔR*^2^ = 0.12; *p* < 0.001). The final model suggested that age (β = −0.07; *p* < 0.05), the ability to express emotions (β = 0.19; *p* < 0.001) and manage emotional states (β = 0.16; *p* < 0.001), in addition to self-esteem (β = 0.44; *p* < 0.001) significantly predict levels of life satisfaction a positive way.

Finally, following the same modus operandi in the prediction of affect balance, the model was established in three steps (*R*^2^ = 0.48; *p* < 0.001). In the first step, sex, age, and number of siblings were entered (*ΔR*^2^ = 0.04; *p* < 0.001). In the second step, the abilities to perceive, express, and manage emotions were added to the prediction (*ΔR*^2^ = 0.33; *p* < 0.001). In the third step, self-esteem was entered as predictor (*ΔR*^2^ = 0.12; *p* < 0.001). In the final model, none of the demographic variables appeared to be significant predictors of affect balance. However, perceiving emotions (β = −0.06; *p* < 0.05), expressing emotions (β = 0.10; *p* < 0.001), managing emotions (β = 0.30; *p* < 0.001), and self-esteem (β = 0.42; *p* < 0.001) showed a significant effect on affect balance.

### 3.3. Combined Contribution of Emotional and Personal Predictors of Adjustment and Well-Being

First, the descriptive statistics of the variables under study were calculated, as well as the calibration values (Table 3). The adjustment and subjective well-being based on emotional and personal factors were then analysed using fsQCA models.

#### 3.3.1. Necessary Conditions

The necessary conditions for high emotional symptoms and high behavioural problems were tested. A condition is considered necessary when it must always be present in order for the result concerned to occur. The results showed that none of the variables was a necessary condition, as all the consistency values were under 0.90 [65].

#### 3.3.2. Sufficient Conditions

In the adjustment, the combination of conditions resulting in high levels of emotional symptoms and behavioural problems was analysed (Table 4). According to Eng and Woodside [66], the fsQCA analysis involves two stages. First, a truth table algorithm transforms the fuzzy-set membership scores into a truth table that enumerates all the logically possible combinations of causal conditions and each configuration’s empirical outcome. The consistency cut-off indicates the cut-off point from which each conditions (or variables) of the combination are considered reliable. Ragin (2008) recommends the consistency cut-off is at least above 0.70. Thus, the frequency cut-off in the true table was established as 1 and the consistency cut-off as 0.85 for both emotional symptoms and behavioural problems. The fsQCA analysis then generates three solutions: complex, parsimonious, and intermediate. Each solution is based on a different treatment of the remaining combinations. In few words, the complex solution is the most restrictive and the parsimonious solution is the least restrictive result. The literature suggests focusing on the intermediate solution [65] and therefore the corresponding results are presented here. The solution indicated five combinations of causal conditions which explained 46% of high levels of emotional problems (overall solution coverage = 0.46; overall solution consistency = 0.81) and eight combinations of causal conditions which explained 30% of high level of behavioural problems in adolescents (overall solution coverage = 0.30; overall solution consistency = 0.80). In fsQCA, a model is informative when consistency is above 0.70 [66]. The solutions therefore seemed to be adequate in view of the results obtained. The most important three combinations for high emotional symptoms and high behavioural problems are shown in Table 4.

The first combination explained 37% of high levels of emotional symptoms. This combination to predict high emotional symptoms was the result of the interaction of a low sex score (girl), low emotional expression, low emotional management, and low self-esteem (raw coverage = 0.37; solution consistency = 0.82). The second combination explained 29%, and the third explained 23%. The second combination to predict high emotional problems was the result of the interaction of a low sex score (girl), low emotional perception, low emotional management, and low self-esteem (raw coverage = 0.29; solution consistency = 0.83) and the third was the result of the interaction of a low sex score (girl), high age, low emotional expression, and low self-esteem (raw coverage = 0.23; solution consistency = 0.87). 

Meanwhile, the first and second combinations explained 15% of high levels of behavioural problems. The first combination to predict high behavioural problems was the result of the interaction of high age, a high number of siblings, high emotional perception, high emotional expression, and low self-esteem (raw coverage = 0.15; solution consistency = 0.83); and the second combination was the result of the interaction of high number of siblings, high emotional perception, low emotional expression, low emotional management, and high self-esteem (raw coverage = 0.15; solution consistency = 0.85). The third combination explained 13%, and was the result of the interaction of a high sex score (boy), high number of siblings, low emotional expression, low emotional management, and low self-esteem (raw coverage = 0.13; solution consistency = 0.84).

In the case of subjective well-being, the combination of conditions resulting in high levels of life satisfaction and high levels of affect balance were analysed (Table 5). In the first stage, a truth table algorithm transforms the fuzzy-set membership scores into a truth table that lists all the logically possible combinations of causal conditions and each configuration’s empirical outcome. The frequency cut-off in the true table was established as 1 and the consistency cut-off as 0.90 for both life satisfaction and affect balance. As in the adjustment model, the fsQCA analysis then generates three possible solutions, and the intermediate solution is presented here.

The intermediate solution indicated seven combinations of causal conditions, which explained 48% of the high level of life satisfaction (overall solution coverage = 0.48; overall solution consistency = 0.89) and nine combinations of causal conditions, which explained 61% of the high level of affect balance in adolescents (overall solution coverage = 0.61; overall solution consistency = 0.83). The models seemed to be adequate, as the consistency of all solutions is over 0.70. The three most important combinations for high life satisfaction and high affect balance are shown in Table 5.

On the one hand, the first combination explained 28% of high levels of life satisfaction. This combination for predicting high levels of life satisfaction was the result of the interaction of a high sex score (boy), high emotional expression, high emotional management, and high self-esteem (raw coverage = 0.28; solution consistency = 0.89). The second and third combinations explained 24%. The second combination for predicting high levels of life satisfaction was the result of the interaction of a high age, low number of siblings, high emotional expression, high emotional management, and high self-esteem (raw coverage = 0.24; solution consistency = 0.90), and the third was the result of the interaction of low age, high number of siblings, high emotional expression, high emotional management, and high self-esteem (raw coverage = 0.24; solution consistency = 0.92).

On the other hand, the first combination explained 42% of high levels of affect balance. The first combination to predict high affect balance was the result of the interaction of a low number of siblings, high emotional perception, high emotional management, and high self-esteem (raw coverage = 0.42; solution consistency = 0.90). The second explicated 30% and the combination predicting a high affect balance was the result of the interaction of a high sex score (boy), high emotional expression, and high self-esteem (raw coverage = 0.30; solution consistency = 0.83). The third combination explained 27% of high levels of affect balance and the combination predicting a high affect balance was the result of the interaction of low age, high number of siblings, high emotional expression, and high self-esteem (raw coverage = 0.27; solution consistency = 0.93).

## 4. Discussion

The aim of this study was to examine the combined contribution of socio-emotional factors (emotional competence and self-esteem) and personal factors (sex, age, and number of siblings) to the prediction of psychological adjustment and subjective well-being in adolescence.

Based on previous research [27,35,36], we expected that higher emotional ability and self-esteem could predict fewer behavioural problems and emotional symptoms, indicating better adjustment. The results from the hierarchical regression and fsQCA models support the first hypothesis, showing a significant influence of emotional abilities and self-esteem on psychological adjustment. For emotional symptoms, the results from both methodologies indicate that the most relevant socio-emotional predictors are self-esteem and emotional management. The regression model suggests that adolescents who pay more attention to their feelings are less able to manage their emotional states, and experience more emotional symptoms. In the results obtained with fsQCA, the combination of adolescents’ low self-esteem, low capacity to express their feelings, and poor ability to manage their emotional states effectively may lead to the development of emotional symptoms. Paying too much attention to one’s own emotions, if not accompanied by adequate emotional expression and regulation skills, may therefore have negative effects on psychological adjustment during adolescence [67].

With regard to behavioural problems, the predictive capacity of social-emotional factors is lower than for emotional symptoms, regardless of the statistical analyses used. Self-esteem seems to be the most relevant socio-emotional predictor of behavioural problems. However, the interplay of these factors is unclear; perhaps that is why the predictive capacity is lower than in emotional symptoms. On the one hand, the regression model indicates that adolescents with a poorer capacity of emotional management and less self-esteem present more behavioural problems. On the other hand, fsQCA analysis suggests that there are several ways to predict the development of behavioural problems. One of the main pathways shows that adolescents who perceive and express their emotions but have low self-esteem develop behavioural problems. Another pathway suggests that adolescents may perceive their emotions accurately and value themselves, but they are less able to express their feelings and manage them ineffectively, developing conduct problems. A third pathway combines adolescents’ low self-esteem with their poor capacity for expressing and managing emotions, predicting behavioural problems. These different pathways seem to indicate that the combination of low self-esteem and low emotional competence, including emotional perception, expression, and management, predict the development of behavioural problems. The role of emotional perception in behavioural problems is critical, given that our findings are contrary to our expectations. Our results therefore suggest that in the absence of the capacity to express and manage emotional states effectively, an increased level of emotional perception may be diminishing adolescents’ adjustment. However, our findings are consistent with previous studies, which provided evidence that paying too much attention to one’s own feelings may increase problematic behaviours such as alcohol consumption in adolescents [68].

With regard to the second hypothesis, we expected that higher levels of emotional ability and self-esteem would predict higher life satisfaction and affect balance, indicating more well-being [28,52]. In general, the results obtained in our study support this hypothesis, suggesting that social-emotional factors positively influence adolescents’ subjective well-being. In the prediction of the cognitive dimension of subjective well-being (life satisfaction), both methodologies acknowledge the influence of emotional expression and management as well as self-esteem. In fact, the latter is the predictor with the highest weight. Adolescents with the greatest capacity to express their feelings, who perceive themselves as more competent to manage their own emotional states and whose self-esteem is higher, tend to be more satisfied with their lives. In the prediction of the affective dimension of subjective well-being (affect balance), self-esteem is the socio-emotional predictor with the most weight in both the regression and the fsQCA models. The results of the hierarchical regression show that adolescents who do not pay much attention to their feelings, but express and manage their emotional states effectively, in addition to high self-esteem, experience more positive than negative effects. These unexpected results for emotional perception are consistent with previous research [69], and suggest that increased attention to emotions and negative feelings could diminish adolescents’ well-being. Moderate or even lower levels of perceiving emotions therefore seem most beneficial for an adolescent’s adjustment and overall well-being. The results from fsQCA indicate that the combination of high self-esteem and high emotional competence (perceive, express, understand, and manage emotions) predicts affect balance in adolescents. In overall terms, emotional expression, emotional management, and self-esteem are the most powerful social-emotional predictors of life satisfaction (cognitive dimension) and affect balance (affective dimension).

The third hypothesis addressed the impact of personal factors, including sex and age, suggesting that girls would experience more emotional symptoms than boys, whereas boys might report more behavioural problems than girls [44,48]. We also expected younger adolescents to be better adjusted and have higher levels of well-being than the older ones [70]. Our results support this hypothesis in terms of the adolescents’ adjustment. More specifically, both regression and fsQCA analyses show that girls tend to present more emotional symptoms than boys. These findings are in line with previous studies, which indicated that girls may have more persistent thoughts or worries and therefore internalise more emotional symptoms [45]. In behavioural problems, sex seems to have some influence, but to a lesser extent than emotional symptoms. An analysis of the results from hierarchical regression and fsQCA revealed that being a boy appears as a predictor of behavioural problems. The influence of age on adolescent adjustment was shown by both methodologies, but was modest in both cases. In the regression model, older adolescents report more emotional problems, but no more behavioural problems than younger ones. Furthermore, in the fsQCA model, the main pathways indicate that older teenagers reported more emotional symptoms and more behavioural problems when combined with deficits in social-emotional competencies. Our findings suggest different patterns for female and male and younger and older adolescents in terms of psychological adjustment. The possible propensity of each adolescent must be addressed, considering that girls are more likely to internalise emotional symptoms, and that boys more likely to externalise behavioural problems, and that emotional and behavioural problems increase with age.

As regards the impact of personal factors (sex and age) on subjective well-being, the results of this study are less conclusive. According to the results from the hierarchical regression, younger adolescents report higher levels of life satisfaction, while no significant effects are observed in the affective dimension. However, sex and age appear in the main combinations of fsQCA models when predicting well-being. Boys seem to report higher levels of subjective well-being than girls (both cognitive and affective dimensions). Similarly, younger adolescents experience more positive rather than negative emotions, in combination with social-emotional factors. The results from the cognitive dimension must be interpreted in terms the number of siblings. Adolescents who are older but have fewer siblings therefore appear to be more satisfied with their lives, as well as those who are younger but have more siblings. These results appear to suggest that the combination of personal factors may provide information complementary to that obtained from linear regression models.

Finally, the fourth hypothesis proposed that the number of siblings would be associated with better adjustment [51]. The results obtained in this study do not confirm this hypothesis. In fact, the regression and fsQCA models show that more siblings are associated with more behaviour problems. However, this unexpected finding is consistent with other results observed in children. One of the studies carried out by Brody [49] suggests that parents’ differential treatment in parenting might be associated with behavioural problems in children, and even with the development of antisocial behaviour. However, interactions with older siblings may promote young children’s linguistic and cognitive development. Further studies that assess the relationship between parents and adolescents are therefore necessary.

Our findings stress the influence of social-emotional factors in the prediction of emotional symptoms and behavioural problems, and despite the subtle differences between them, both arise above all from a deficit in managing emotions and low self-esteem. Some adolescent adjustment problems, especially emotional symptoms, could therefore be prevented and reduced by improving the social-emotional skills of young people. Furthermore, in addition to high levels of self-esteem and a high capacity for emotional management, the ability for emotional expression is also necessary in order to achieve high levels of well-being. Intervention programs that focus on the development of social-emotional competencies should therefore foster self-esteem and focus especially on the management and expression of emotions to prevent the onset of adjustment problems, as well as to improve subjective well-being during adolescence [32,33,34].

In summary, the results of both methodologies (regression and fsQCA models) indicate that socio-emotional and personal factors influence adolescent adjustment and well-being. On the one hand, older adolescents, especially girls, with a low capacity to manage their emotional states and low self-esteem tend to internalise more emotional symptoms. Meanwhile, adolescents, especially boys, with more siblings, with a low capacity to manage their emotional states and low self-esteem tend to externalise more behavioural problems. On the other hand, adolescents with greater capacity to express and manage their emotions and more self-esteem are more satisfied with their lives. Age also seems to be an influence in combination with the number of siblings: older adolescents and those with fewer siblings, or younger teenagers with more siblings evaluate their lives more positively. Finally, teens who express their moods and manage their emotional states effectively in addition to having higher self-esteem tend to experience positive rather than negative emotions more often.

One of the strengths of this study was its methodological approach. Comparing both methodologies in the prediction of adolescent adjustment and well-being, fsQCA models include a greater number of factors than regression models. In addition, fsQCA offers a variety of pathways where predictors can be combined in different ways, depending on the relations between variables. fsQCA methodology is therefore an analysis method that could complement traditional regression models. There are some limitations in the study that need to be pointed out. First, the sample was obtained by convenience sampling, which makes it difficult to generalize the results obtained. In future research, it would be advisable to carry out a stratified random cluster sampling that includes adolescents from all over the country. Second, the variables included in the study must be reported by the adolescents themselves, which makes them inherently subjective. However, in future research it would be useful to include multiple reports from parents, teachers and peers, who would provide information about the adolescents studied. Third, the data might be constrained by participants who responded in a random, pseudorandom, or dishonest manner. Hence, the results obtained in the research may not be entirely reliable. In future research, infrequency scales will be incorporated, as they do in other studies with adolescents and young people [71].

Despite the limitations of this study, this research makes a contribution to the study of potential risk and protective factors for psychological adjustment and subjective well-being in adolescence. Furthermore, this study provides valuable insights for practical applications. First, a lack of self-esteem and emotional management may increase the development of both emotional symptoms and behavioural problems, indicating poor adjustment. Similarly, these social-emotional factors, including emotional expression, promote subjective well-being when they are well developed. Focusing intervention programs solely on the development of self-esteem and emotional management may therefore prevent emotional and behavioural problems. Socio-emotional training aimed at promoting well-being should nevertheless include emotional expression. Second, personal factors must be taken into account in order to tailor the prevention or intervention program to each target group. The results of the study suggest that girls will probably suffer from more emotional symptoms, while boys will experience more behavioural problems. Likewise, it seems that more adjustment problems may appear with age, and the number of siblings has a relevant influence on behavioural problems: the more siblings, the more problems.

## 5. Conclusions

Adolescence is a period of change in almost every aspect of adolescent life that may impact their adjustment and well-being. However, it should not only be understood as a period of vulnerability, but also as a time of opportunity due to adolescents’ neural plasticity. The agents who interact with adolescents, e.g., psychologists, doctors, educators, fathers and mothers, play a fundamental role in this stage. They should be aware that social-emotional and personal factors influence adolescents’ health. Our research contributes to an in-depth understanding of the socio-emotional and personal factors that influence adjustment and well-being, and the way they affect adolescents individually and as a combination. These findings enable intervention programs that aim to promote psychological adjustment and well-being to be improved. In addition to the development of social-emotional competencies and paying attention to personal factors, intervention programs also need to focus on getting adolescents involved and motivated to participate. Digital tools such as mobile applications and online platforms are being incorporated in several health fields such as nutrition, physical exercise, and medicine. These technological devices allow us to reach out to adolescents who belong to a digital generation that relates to the world through technology. Incorporating technologies into social-emotional learning programmes to promote psychological adjustment and well-being in adolescence may be beneficial for all these reasons.

## Figures and Tables

**Table 1:** Bivariate correlations among variables studied.

Variables	1	2	3	4	5	6	7	8	9
1. Age	-								
2. Perceive emotions	−0.02	-							
3. Express emotions	−0.07 *	0.39 **	-						
4. Manage emotions	−0.07 *	0.33 **	0.57 **	-					
5. Self-esteem	−0.15 **	0.17 **	0.44 **	0.56 **	-				
6. Emotional symptoms	0.16 **	0.01	−0.26 **	−0.37 **	−0.58 **	-			
7. Conduct problems	0.09 **	−0.07 *	−0.14 **	−0.22 **	−0.28 **	0.29 **	-		
8. Life satisfaction	−0.16 **	0.22 **	0.48 **	0.52 **	0.62 **	−0.42 **	−0.29 **	-	
9. Affect balance	−0.13 **	0.15 **	0.44 **	0.58 **	0.63 **	−0.53 **	−0.26 **	0.59 **	-

* *p* < 0.05. ** *p* < 0.01.

**Table 2:** Hierarchical Multiple Regression Analyses.

Predictor	Emotional Symptoms				Behavioural Problems				Life Satisfaction				Affect Balance			
	*ΔR* ^2^	*ΔF*	ß	*t*	*ΔR* ^2^	*ΔF*	ß	*t*	*ΔR* ^2^	*ΔF*	ß	*t*	*ΔR* ^2^	*ΔF*	ß	*t*
Step 1	0.09	30.08 ***			0.02	6.06 ***			0.04	11.94 ***			0.04	12.91 ***		
Sex			−0.26	−8.05 ***			0.08	2.30 **			0.13	3.76 ***			0.17	4.92 ***
Age			0.15	4.45 ***			0.09	2.75 *			−0.15	−4.41 ***			−0.12	−3.48 ***
Number of siblings			0.03	1.02			0.08	2.36 **			−0.01	−0.38			−0.02	−0.49
Step 2	0.12	42.40 ***			0.05	15.68 ***			0.29	121.428 ***			0.33	144.21 ***		
Perceive emotions			0.12	3.55 ***			0.04	1.03			0.00	0.10			−0.07	−2.26 **
Express emotions			−0.09	−2.20 **			−0.04	−0.86			0.27	7.40 ***			0.18	5.10 ***
Manage emotions			−0.33	−8.63 ***			−0.22	−5.39 ***			0.35	10.00 ***			0.49	14.33 ***
Step 3	0.16	219.81 ***			0.04	34.71 ***			0.12	190.74 ***			0.12	186.97 ***		
Self-esteem			−0.50	−14.83 ***			−0.24	−5.89 ***			0.44	13.81 ***			0.42	13.67 ***
*Dubin*–*Watson*	2.10				2.03				1.96				1.93			
*R* ^2^	0.38 ***				0.10 ***				0.45 ***				0.48 ***			

*ΔR*^2^*=* change in *R*^2^; *ΔF =* change in *F*; ß = regression coefficient; *t* = value of *t*-test statistic; * *p* < 0.05. ** *p* < 0.01. *** *p* < 0.001.

**Table 3:** Descriptive statistics and calibration values.

Descriptive Statistics		PE	EE	ME	SE	ES	BP	LS	AB
Mean		50,307.34	45,116.42	45,581.17	159,191.89	26.26	9.19	3865.30	3478.74
Standard deviation		53,785.59	63,457.50	54,658.04	243,618.83	41.04	15.87	4259.07	4214.42
Minimum		1.00	1.00	1.00	4.00	1.00	1.00	1.00	−15,624.00
Maximum		279,936.00	279,936.00	279,936.00	1,048,576.00	243.00	243.00	16,807.00	15,624.00
Calibration values									
Percentile	10	2602.80	216.00	1512.00	777.60	2.00	1.00	96.00	−288.00
	50	32,200.00	18,000.00	24,000.00	52,488.00	8.00	4.00	2160.00	2748.00
	90	129,600.00	135,000.00	112,500.00	516,096.00	72.00	24.00	10,290.00	9784.00

PE = perceive emotions. EE = express emotions. ME = manage emotions. SE = self-esteem. ES = emotional symptoms. BP = behavioural problems. LS = life satisfaction. AB = affect balance.

**Table 4:** Combinations from intermediate solution for adjustment.

Frequency Cutoff: 1	High Emotional Symptoms			High Behavioural Problems		
	Consistency Cut-off: 0.85			Consistency Cut-off: 0.85		
	1	2	3	1	2	3
Sex	○	○	○			●
Age			●	●		
Number of siblings				●	●	●
Perceive emotions		○		●	●	
Express emotions	○		○	●	○	○
Manage emotions	○	○			○	○
Self-esteem	○	○	○	○	●	○
Consistency	0.82	0.83	0.87	0.83	0.85	0.84
Raw coverage	0.37	0.29	0.23	0.15	0.15	0.13
Unique coverage	0.050	0.013	0.006	0.044	0.033	0.013
Overall Solution Consistency			0.81			0.80
Overall Solution Coverage			0.46			0.30

● = high levels, ○ = low levels, empty space = the condition is not present in the combination. The numbers (1, 2, 3) represent the first, second, and third principal combinations. All sufficient conditions are adequate.

**Table 5:** Combinations from intermediate solution for subjective well-being.

Frequency Cutoff: 1	High Life Satisfaction			High Affect Balance		
	Consistency Cut-off: 0.90			Consistency Cut-off: 0.90		
	1	2	3	1	2	3
Sex	●				●	
Age		●	○			○
Number of siblings		○	●	○		●
Perceive emotions				●		
Express emotions	●	●	●		●	●
Manage emotions	●	●	●	●		
Self-esteem	●	●	●	●	●	●
Consistency	0.89	0.90	0.92	0.90	0.83	0.93
Raw coverage	0.28	0.24	0.24	0.42	0.30	0.27
Unique coverage	0.033	0.043	0.039	0.000	0.060	0.015
Overall Solution Consistency			0.89			0.83
Overall Solution Coverage			0.48			0.61

● = high levels, ○ = low levels, empty space = the condition is not present in the combination. The numbers (1, 2, 3) represent the first, second, and third principal combinations. All sufficient conditions are adequate.
